# Supplementary material for: Systematic Review with Meta-Analysis: Comparison of the Risk of Hepatocellular Carcinoma in Antiviral-Naive Chronic Hepatitis B Patients Treated with Entecavir versus Tenofovir: The Devil in the Detail
Source: Cancers (Basel). 2022 May 25;14(11):2617. doi: 10.3390/cancers14112617 (PMC9179302; doi:10.3390/cancers14112617)
Supplement: Supplementary file 1 [file cancers-14-02617-s001.zip › cancers-1657632-supplementary.pdf]

cancers

MDPI

Supplementary Material: Systematic Review with Meta-Analysis: Comparison of the Risk of Hepatocellular Carcinoma in Antiviral-Naive Chronic Hepatitis B Patients Treated with Entecavir versus Tenofovir: The Devil in the Detail

Hyunwoo Oh, Hyo Young Lee, Jihye Kim and Yoon Jun Kim

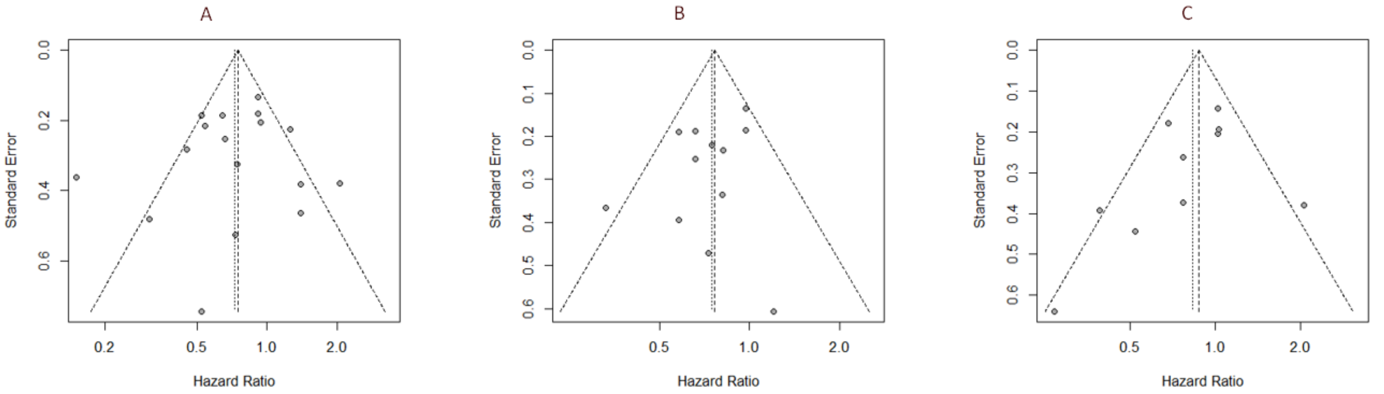

Figure S1. Analysis of publication bias. Using the AS-Thompson's test for publication bias, no significant asymmetry is seen in the funnel plots ($P > 0.1$) for Pooled analysis of representative HRs presented in individual papers (**A**), adjusted HR (**B**), and PS-matched HR (**C**).

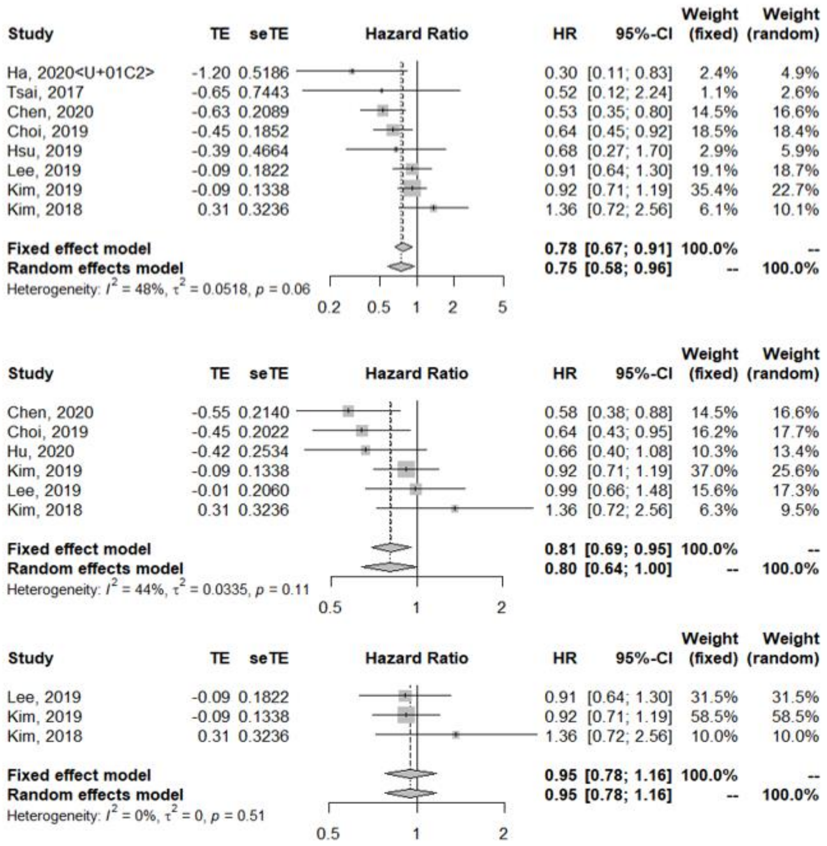

Figure S2. Pooled analysis of representative HRs presented in individual papers/multivariable-adjusted HR/propensity score-matched HR in the cirrhotic subcohort.

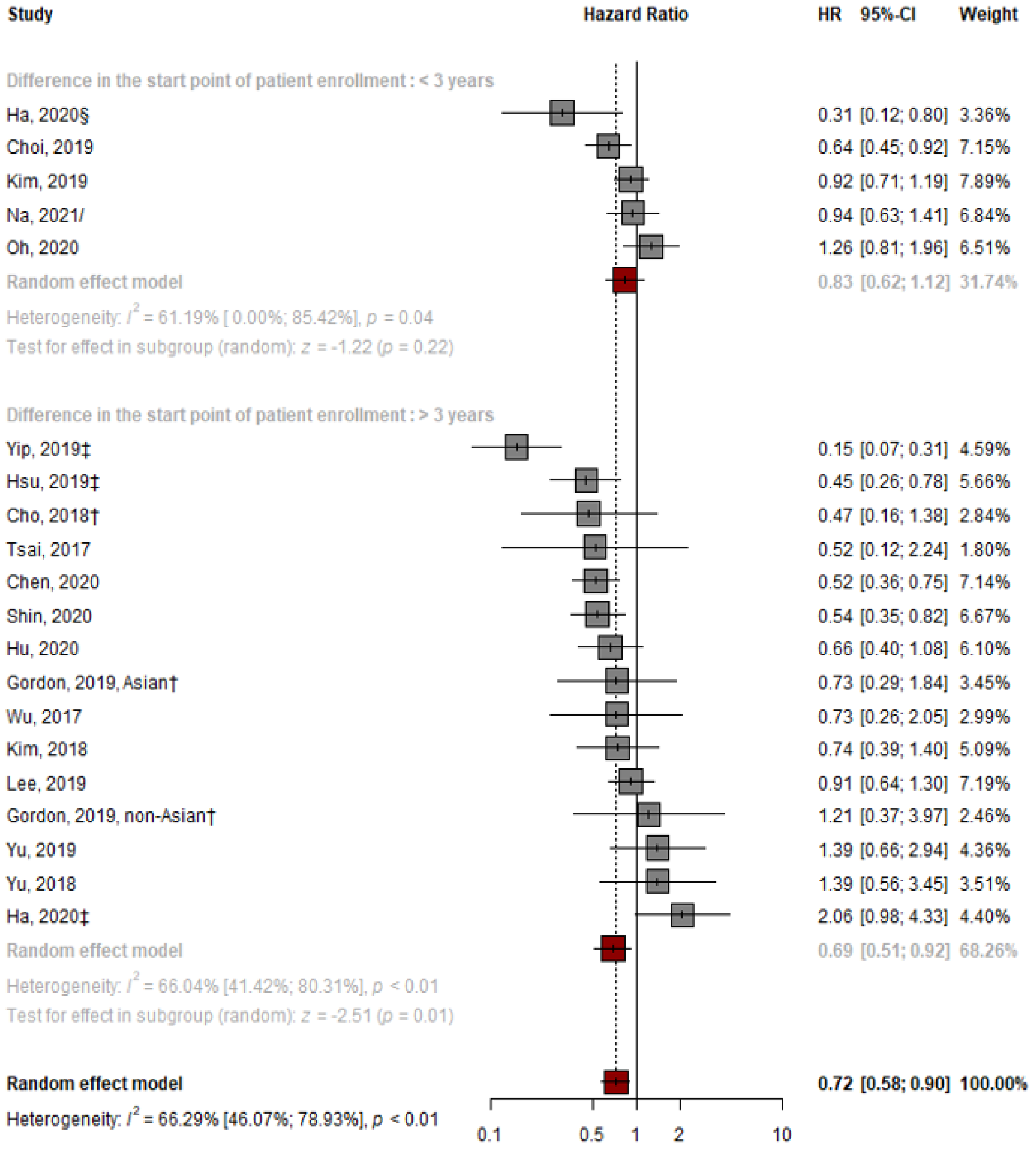

Figure S3. Pooled HR from a subgroup analysis according to the starting point. † abstract; ‡ suggest outcomes from competing risk analysis; § Ha from CHA Bundang Medical Center, CHA University; / from unadjusted cohort at the time of CVR.

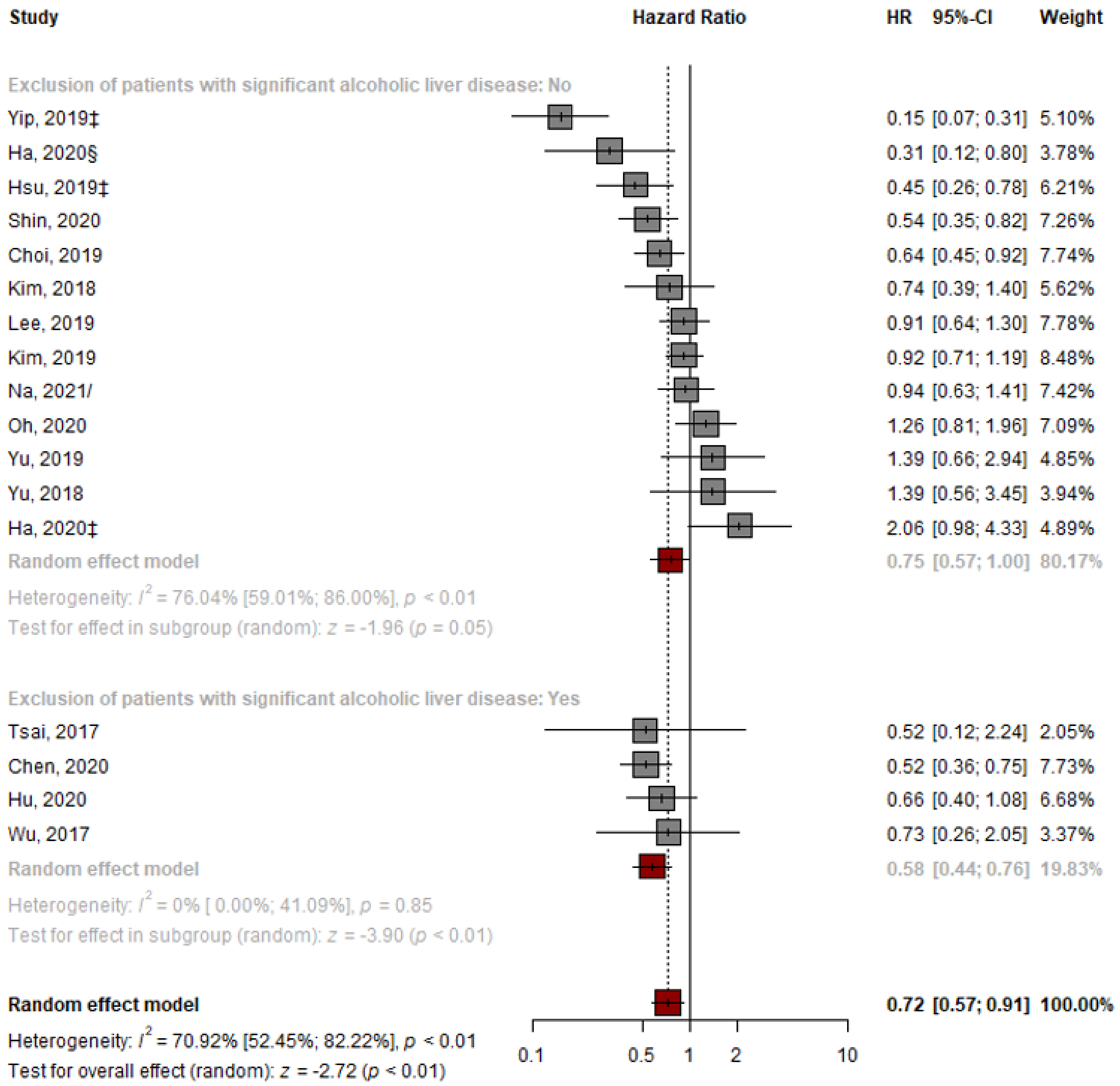

Figure S4. Pooled HR from a subgroup analysis after excluding alcoholic liver disease. ‡ suggest outcomes from competing risk analysis; § Ha from CHA Bundang Medical Center, CHA University; / from unadjusted cohort at the time of CVR.

Table S1. Search strategies.

Data Base	Search Strategy
Medline	1. (tenofovir) OR (tenofovir [tw])<br>2. (entecavir) OR (entecavir [tw])<br>3. (hepatocellular carcinoma) OR (hepatocellular carcinoma [tw])<br>4. 1 AND 2<br>5. 3 AND 4
EMBASE	1. ('tenofovir'/exp OR 'tenofovir':ab,ti OR 'tenofovir'/de) AND [embase]/lim<br>2. ('entecavir'/exp OR 'entecavir':ab,ti OR 'entecavir'/de) AND [embase]/lim<br>3. ('hepatocellular carcinoma'/exp OR 'hepatocellular carcinoma':ab,ti OR 'hepatocellular carcinoma'/de) AND [embase]/lim<br>4. 1 AND 2<br>5. 3 AND 4
Cochrane	1. tenofovir:ti,ab,kw<br>2. entecavir:ti,ab,kw<br>3. hepatocellular carcinoma:ti,ab,kw<br>4. 1 AND 2<br>5. 3 AND 4

Table S2. Newcastle-Ottawa scale for non-randomized studies (Abstracts were excluded from assessment.).

Bias	Choi 2019	Kim 2018	Shin 2020	Kim 2019	Lee 2019	Tsai 2017	Yip ‡ 2019	Yu 2018	Yu 2019	Wu 2017	Hsu ‡ 2019	Ha § 2020	Oh 2020	Ha ‡ 2020	Hu 2020	Chen 2020	Na / 2021
Selection																	
1) Representativeness of the exposed cohort	★	★	★	★	★	★	★	★	★	★	★	★	★	★	★	★	★
2) Selection of the non exposed cohort	★	★	★	★	★	★	★	★	★	★	★	★	★	★	★	★	★
3) Ascertainment of exposure	★	★	★	★	★	★	★	★	★	★	★	★	★	★	★	★	★
4) Demonstration that outcome of interest was not present at start of study	★	★	★	★	★	★	★	★	★	★	★	★	★	★	★	★	★
Comparability																	
1) Comparability of cohorts on the basis of the design or analysis	★	★	★	★	★	★	★	★	★	★	★	★	★	★	★	★	★
2) Comparability of cohorts on the control of variables	★	★	★	★	★	★	★	-	-	-	★	★	★	★	★	★	★
Outcome																	
1) Assessment of outcome	★	★	★	★	★	★	★	★	★	★	★	★	★	★	★	★	★
2) Was follow-up long enough for outcomes to occur (mean ≥ 5 yrs, each arm)	-	-	-	-	-	-	-	-	-	-	-	-	-	-	★	-	-
3) Adequacy of follow up of cohort (Follow up rate ≥ 80%)	-	-	-	-	-	-	-	-	-	-	-	-	★	-	-	-	-

‡ suggest outcomes from competing risk analysis; § Ha from CHA Bundang Medical Center, CHA University; / from unadjusted cohort at the time of CVR. -, The research isn't according with this point, based on the Newcastle-Ottawa scale; ★, The research is according with this point, based on the Newcastle-Ottawa scale.

Table S3. Adjusted variables for Cox regression analyses for risk of HCC development in the included articles.

Author Year	Age	Sex	Cirrosis	HBeAg	HBV_DNA	AST	ALT	Alb	Bil	Cr	AFP	PT	PLT	DM	HTN	Additional variables
Choi 2019	●	●	●	●	●		Log *	●				●	●	●		Ascites *, CTP, Virologic response (HBV-DNA < 60 at 1 year of treatment) *
Kim, 2018	●	●	●	●		●	●	●					●	●		Duration of treatment *
Shin, 2020	●	●	●	●	●		●	●				●	●	●		Adherence *, VR12 *, MVR *
Kim 2019																NA
Lee 2019	●	○	○	○	●	○	○	○	○	○	○	○	●	●	●	Alcohol, BMI, APRI, FIB-4 *, CTP, Varix (Y/N), GGT, Treatment initiation
Tsai 2017	●	○		○	●			○	○	○	○		○	○	○	BMI, Metformin use, Statin use *, Anti-PLT use, HBV genotype, HBsAg titer, CTP, Ascites, Variceal bleeding (Y/N) *, MELD, Virologic response
Yip 2019 ‡	●	●	●	●	○		●	●	○			○	●	○	○	Enrolled calendar year of patients *
Yu 2018	●	●	●	○	○		○	●	●		○	●	○			Virologic response (HBV-DNA negative at 12 months of AVT)
Yu 2019	●	●	●	○	○		○	●	○		○	○	○			Suboptimal response/virologic failure after AVT
Wu 2017	○	○	○	●	●	○	○		○	○	○		○	○	○	FIB-4, HBsAg titer *, HBV Genotype
Hsu 2019 ‡	●	●	●	○	○	○	○	●	○	○	●	○	●	●		Region *, Decompensation, BMI, FIB-4
Ha 2020 §	○	○	○	○	○		○	○	○	○		○	○	○		BMI, CTP, Biochemical/Serological/Virological response, SVR
Oh 2020	●	○	●	●	●			●	○	GFR	○	●	●	○	●	CKD
Ha 2020 ‡	●	●	○	○	●	○	○	●	○		○	○	●	○		Alcohol, HBsAg titer
Hu 2020	○		-		○		○	○			●		●	●		FIB-4, HCC family history *, Varix *
Chen 2020	●	●	-	○	○	○	○	●	○	GFR	○	○	●	●	○	(NA experience) Decompensation *, CTP, FIB-4, APRI
Na 2021 /	●	●	●	○	●		●	●	●	GFR	●	●	●	●		Time to CVR, Cholesterol *

○ ; variables for univariate Cox regression only, ●, * ; variables for multivariate Cox regression; ‡ suggest outcomes from competing risk analysis; § Because of the low number of events (HCC), authors did not performed multivariable analyses; / from unadjusted cohort at the time of CVR Cr, creatinine; GFR, glomerulus filtration rate; AST, aspartate aminotransferase; ALT, alanine aminotransferase; PT, prothrombin time; PLT, platelet; AFP, alpha fetoprotein; BMI, body mass index; APRI, AST/platelet ratio index; FIB-4, fibrosis-4 index; CTP, Child-Turoctte-Pugh; MELD, model for end-stage liver disease; GGT, gamma-glutamyl transferase; VR12, virologic response at 12 months; MVR, maintained virologic response; SVR, sustained virologic response; CVR, complete virologic response; DM, diabetes mellitus; HTN, hypertension; CKD, chronic kidney disease; NA, Nucleos(t)ide analogue;

Table S4. Adopted variables for propensity score matching analysis to reduce selection bias and the effect of potential confounders in the included articles.

Author Year	Age	Sex	Cirrosis	HBeAg	HBV_DNA	AST	ALT	Alb	Bil	Cr	AFP	PT	PLT	DM	HTN	Additional Variables
Choi 2019	○	○	○	○	○		○	○	○	○		○	○	○	○	Ascites, CTP, CU-HCC, GAG-HCC, PAGE-B, REACH-B
Kim, 2018	○	○	○	○	○	○	○	○	○	○	○	○	○	○	○	
Shin, 2020	○	○	○	○	○		○	○	○	○		○	○	○	○	GAG-HCC, CU-HCC, REACH-B, PAGE-B
Kim 2019	○	○	○	○				○	○				○	○	○	
Lee 2019	○	○		○	○	○	○	○	○	○	○	○	○	○	○	(severity of underlying liver disease), Alcohol, BMI, APRI, FIB-4, CTP, Varix (Y/N), GGT
Yip 2019‡	○	○	○	○	○		○	○	○	○		○	○	○	○	Ascites, Encephalopathy, Renal replacement therapy, Enrolled calendar year of patients
Wu 2017	○		○	○	○											
Hsu 2019 ‡	○	○	○	○	○		○						○	○		Country, Decompensation
Ha 2020 §	○	○	○	○	○		○	○	○	○		○	○	○		CTP, GAG-HCC, CU-HCC, PAGE-B, SVR
Oh 2020	○	○	○	○	○			○	○	GFR	○	○	○	○	○	CKD, Decompensation, CTP, MELD, FIB-4
Ha 2020 ‡	○	○	○	○	○	○	○	○	○		○	○	○	○		Alcohol, Enrolled calendar year of patients, HBsAg titer
Hu 2020	○		-				○	○			○					
Chen 2020	○	○	-	○	○	○	○	○	○	GFR	○	○	○	○	○	(NA experience) Decompensation, FIB-4, APRI
Na 2021 /	○	○	○	○	○		○	○		GFR		○	○			Time to CVR, Cholesterol

AST, aspartate aminotransferase; ALT, alanine aminotransferase; PT, prothrombin time; PLT, platelet; AFP, alpha fetoprotein; BMI, body mass index; APRI, AST/platelet ratio index; FIB-4, fibrosis-4 index; CTP, Child-Turoctte-Pugh; MELD, model for end-stage liver disease; GGT, gamma-glutamyl transferase; VR12, virologic response at 12 months; MVR, maintained virologic response; SVR, sustained virologic response; DM, diabetes mellitus; HTN, hypertension; CKD, chronic kidney disease; CU-HCC, Chinese University HCC score; GAG-HCC, Guide With Age, Gender, HBV DNA, Core Promoter Mutations, and Cirrhosis–HCC score; PAGE-B, platelet age gender B score; REACH-B, Risk Estimation for Hepatocellular Carcinoma in Chronic Hepatitis B score; ‡ suggest outcomes from competing risk analysis; § Ha from CHA Bundang Medical Center, CHA University; / from unadjusted cohort at the time of CVR.

Table S5. Statistical methods used in the included articles.

Author Year	Adopted Method for Variable Selection in Cox Regression	Cut Off of *P* Value to Select the Variables for Multivariate Model	Propensity Score Matching Method	IPTW	Competing Risk Analysis (Model by Fine and Gray)	Multiple Imputation for Missing Data
Choi 2019	-	NA	Nearest-neighbor 1:1 matching Caliper size of 0.1	○	○	0.02% to 3.7%
Kim, 2018	-	0.2	Caliper size of 0.2	-	-	-
Shin, 2020	-	-	Caliper size of 0.1	○	○	○
Kim 2019	-	-	○	○	-	-
Lee 2019	(Sandwich covariance matrix estimation)	-	Nearest-neighbor 1:1 matching Caliper size of 0.2	○	○	0% to 4.6%
Tsai 2017	Stepwise method	-	-	-	-	-
Yip 2019 ‡	Backward elimination	-	Nearest-neighbor 1:1 matching	○	○	HBeAg status (19.7%) HBV DNA level (35.7%) ALT (4.2%) Albumin (4.0%) Total bilirubin (4.5%) INR (21.8%) Platelet (7.2%) Creatinine (10.6%)
Yu 2018	-	-	-	-	-	-
Yu 2019	Backward deletion	-	-	-	-	-
Wu 2017	-	-	Nearest available matching Caliper size NA	-	-	-
Hsu 2019 ‡	Stepwise approach	-	Caliper 0.2	-	○	-
Ha 2020 §	-	-	○	○	-	Platelet (2.5%) Albumin (1.7%) Total bilirubin (1.2%) PT (2.2%) Creatinine (1.7%)
Oh 2020	-	-	Nearest-neighbor 1:1 matching Caliper size NA	-	-	-
Ha 2020 ‡	-	-	Nearest-neighbor 1:1 matching Caliper size NA	○	○	-
Hu 2020	Stepwise selection	-	Caliper size of 0.15 and 0.2	○	-	No missing data
Chen 2020	Forward method	0.25	Nearest-neighbor 1:1 matching Caliper size of 0.2	○	-	○
Na 2021 /	-	-	Nearest-neighbor 1:1 matching Caliper size of 0.1	○	-	eGFR in 7 (0.5%) platelet 19 (1.4%) HBeAg in 47 (3.5%) PT in 51 (3.8%) HBV DNA in 63 (4.7%)

‡ suggest outcomes from competing risk analysis; § Ha from CHA Bundang Medical Center, CHA University; / from unadjusted cohort at the time of CVR., ○, authors used statistical techniques in first row, IPTW, Inverse Probability Treatment Weighting; NA, not available.

Table S6. Characteristics after propensity score matching analysis in the included studies.

Author Year Country	Cirrhosis (%)	Patients (*n*) TDF ETV	Age (mean (± SD)) TDF ETV	Sex (Male%) TDF ETV	HBV_DNA (log10) (IU/mL)	HBeAg Positive (%)
Choi 2019	505 (58.1)	869	48.8 ± 10.4	540 (62.1)	6.5 (5.6, 7.7)	481 (55.4)
Korea	511 (58.8)	869	48.8 ± 10.4	519 (59.7)	6.5 (5.3, 7.7)	479 (55.1)
Kim, 2018	156 (44.1)	354	50 ± 11	223 (63.0)	6.2 ± 1.5	223 (63.0)
Korea	169 (47.7)	354	50 ± 11	220 (62.1)	6.2 ± 1.4	232 (65.5)
Shin, 2020	282 (47.88)	589	50 ± 11	358 (60.8)	6.22 (4.99–7.63)	354 (60.1)
Korea	276 (46.86)	589	50 ± 11	365 (320.)	6.11 (4.99–7.44)	365 (61.97)
Kim 2019	400 (31.3)	1278	48.2 ± 12.0	913 (64.6)	5.55 ± 2.09	640 (50.1)
Korea	394 (30.8)	1278	48.6 ± 11.4	889 (59.9)	5.62 ± 2.11	640 (50.1)
Lee 2019	464 (33.87)	1370	46.92 (11.13)	798 (58.25)	6.39 (5.34, 7.49)	807 (58.91)
Korea	465 (33.94)	1370	42.9 ± 12.7	806 (58.83)	6.51 (5.30, 7.71)	814 (59.42)
Yip 2019 ‡	37 (3.1)	1200	44.4 ± 13.1	587 (48.9)	4.8 ± 2.7	625 (52.1)
Hongkong	NA (3.6)	4636	42.9 ± 12.7	NA (48.9)	4.8 ± 2.8	NA (53.5)
Wu 2017	29(27.4)	106	47.1 ± 12.1	74 (69.8)	7.35 ± 0.7	50(47.1)
Taiwan	57(26.9)	212	46.3 ± 13.2	230 (73.5)	7.26 ± 0.73	100(47.2)
Hsu 2019 ‡	105 (20.19)	520	44.88 ± 0.55	338 (65.0)	5.07 ± 0.10	177 (34.0)
Worldwide	107 (20.58)	520	44.12 ± 0.54	354 (68.1)	5.0 ± 0.10	187 (36.0)
Ha 2020 ‡	39 (9)	298	48 ± 14	179 (60)	6.29 (2.51)	174 (58)
Korea	39 (9)	298	48 ± 16	181 (61)	6.43 (2.77)	161 (54)
Oh 2020	224 (43.4)	516	49.0 ± 9.4	325 (63.0)	6.4 [5.4, 7.5]	311 (60.3)
Korea	238 (46.1)	516	49.2 ± 12.6	319 (61.8)	6.4 [5.4, 7.5]	314 (60.9)
Ha 2020 §	56 (33.3)	168	45.0 ± 11.6	94 (56.0)	7.74 (6.48, 8.74)	109 (64.9)
Korea	58 (34.5)	168	45.4 ± 10.9	100 (59.5)	7.82 (6.85, 8.64)	111 (66.1)
Hu 2020	100%	157	58.6 ± 11.0	115 (73.2)	NA	28 (17.6)
Taiwan		607	58.8 ± 10.8	442 (72.8)		114 (18.7)
Na 2021 /	299 (52.5)	570	50 (44, 57)	334 (58.6)	5.7 (4.5, 6.8)	202 (35.4)
Korea	299 (52.5)	570	50 (44, 56)	339 (59.5)	5.7 (4.6, 6.8)	177 (31.0)

‡ suggest outcomes from competing risk analysis; § Ha from CHA Bundang Medical Center, CHA University; / from unadjusted cohort at the time of CVR.

Table S7. Reimbursement policies for antiviral therapies.

Nation	Reimbursement Polish for NUC (ETV/TDF)		Year of Commencement of Insurance Benefits for NUC	
			ETV	TDF
Korea	2005-12-14	HBeAg(+/−) : HBV-DNA 10^5 copies/mL, AST/ALT > 80 IU	2007-01-01	2012-12-01
	2010-10-01	HBeAg(+) : HBV-DNA 10^5 copies/mL, AST or ALT > 80 IU<br>HBeAg(−): HBV-DNA 10^4 copies/mL, AST or ALT > 80 IU<br>Cirrhosis/HCC : HBV-DNA > 10^4 copies/mL, AST or ALT > UNL		
	2015.09.01	HBeAg(+) : HBV-DNA 10^5 copies/mL, AST or ALT > 80 IU<br>HBeAg(−) : HBV-DNA 10^4 copies/mL, AST or ALT > 80 IU<br>Compensated cirrhosis: HBV-DNA > 10^4 copies/mL<br>Decompensated cirrhosis/HCC: HBV-DNA(+)		
	2016.05.01	HBeAg(+) : HBV-DNA > 20000 IU/mL, AST or ALT > 80 IU<br>HBeAg(−) : HBV-DNA > 2000 IU/mL, AST or ALT > 80IU		

		Compensated cirrhosis: HBV-DNA > 2000 IU/mL<br>Decompensated cirrhosis/HCC: HBV-DNA(+)		
Taiwan	2003-10-01	HBeAg(+): 18 months,<br>ALT > 5 × UNL or 2 × UNL < ALT < 5 × UNL + HBV DNA>20000 IU/mL	2008.08.01	
	2004-08-01	HBeAg(-) : 18 months,<br>ALT > 2*UNL + HBV DNA > 2000 IU/mL		
	2009-11-01	HBeAg(+) & HBeAg(−) : Extend NUC to 3 years, LC remains lifelong		2011-06-01
	2017-01-01	eAg+: no limitation of treatment duration until eAg loss+1 year consolidation		
	2019-02-01	Curative HCC + HBV DNA > 2000 IU/mL		
Hongkong	2010	According to APASL criteria at that time	2010	2012<br>2nd line or with pregnancy indication
	2013-2014	ALT > 58 U/L.<br>Cirrhosis proven by liver fibrosis or Fibroscan results<br>There is often delays (1-2 years) from APASL guideline update and change in reimbursement criteria.		
Japan		No restriction in reimbursement according to viral and biochemical status	2010	2014
China		(varies from region to region)		
Beijing		HBeAg(+) : HBV-DNA 105 copies/mL, AST or ALT > 2 × ULN<br>HBeAg(−): HBV-DNA 104 copies/mL, AST or ALT > 2 × ULN<br>Cirrhosis/HCC : HBV-DNA positive	2005.12. (Shanghia)<br>2009. (All over China)	2016
Guangdong	2005	HBeAg(+): HBV DNA > 10^5 copies/mL, ALT ≥ 2 ULN or G ≥ 2 or S ≥ 2<br>HBeAg(−) : HBV DNA > 10^4 copies/mL, ALT ≥ 2 ULN or G ≥ 2 or S ≥ 2		
	2010	HBeAg(+): HBV DNA > 10^5 copies/mL or 20,000 IU/mL, ALT ≥ 2ULN or G ≥ 2 or S ≥ 2<br>HBeAg(−): HBV DNA > 10^4 copies/mL or 2000 IU/mL， ALT ≥ 2 ULN or G ≥ 2 or S ≥ 2		
	2015	HBeAg(+): HBV DNA > 20,000 IU/mL, ALT ≥ 2 ULN or G ≥ 2 or S ≥ 2<br>HBeAg(−): HBV DNA > 2000 IU/mL, ALT ≥ 2 ULN or G ≥ 2 or S ≥ 2		
	2019	HBeAg(+): HBV DNA detectable, ALT ≥ 1 ULN or G ≥ 2 or S ≥ 2<br>HBeAg(−): HBV DNA detectable, ALT ≥ 1 ULN or G ≥ 2 or S ≥ 2		

Listed are the reimbursement policies for ETV and TDF in countries in the included articles. ETV: entecavir; TDF: Tenofovir disoproxil fumarate.
